# ESI-MS/MS Analysis of Phenolic Compounds from *Aeonium arboreum* Leaf Extracts and Evaluation of their Antioxidant and Antimicrobial Activities

**DOI:** 10.3390/molecules26144338

**Published:** 2021-07-17

**Authors:** Sahar Affes, Amer Ben Younes, Donyez Frikha, Noureddine Allouche, Michel Treilhou, Nathan Tene, Raoudha Mezghani-Jarraya

**Affiliations:** 1 Laboratory of Organic Chemistry LR17ES08, Natural Substances Team, Faculty of Sciences of Sfax, University of Sfax, P.B. 1171, Sfax 3000, Tunisia; affessahar0@gmail.com (S.A.); amer.benyounes@live.fr (A.B.Y.); noureddineallouche@yahoo.fr (N.A.); 2 Unité Biodiversité et Ecosystèmes Aquatiques Environnementaux (UR11ES/72), Faculté des Sciences de Sfax, Université de Sfax, BP 1171, Sfax 3000, Tunisia; fdonyez@gmail.com; 3 Equipe BTSB-EA 7417, Institut National Universitaire Jean-François Champollion, Université de Toulouse, Place de Verdun, 81012 Albi, France; michel.treilhou@univ-jfc.fr

**Keywords:** *Aeonium arboreum*, LC–MS/MS analysis, phenolic compounds, flavonoids, antifungal activity, antioxidant activity, antimicrobial activity

## Abstract

*Aeonium* is a genus of succulents belonging to the Crassulaceae family. Their importance in traditional medicine has stimulated both pharmacological and chemical research. In this study, we optimized extraction, separation, and analytical conditions using a high performance liquid chromatographic method coupled with electrospray ionization mass spectrometry by the negative mode (HPLC-ESI-MS) in order to, for the first time, determine thirty-four compounds from *Aeonium arboreum* leaves. Twenty-one of them are assigned among which are sixteen flavonoids and five phenolic acids. FRAP, TAC, DPPH, and ABTS^•+^ radical scavenging were used to evaluate antioxidant activity. The obtained IC_50_ values ranged from 0.031 to 0.043 mg.mL^−1^ for DPPH and between 0.048 and 0.09 mg·mL^−1^ for ABTS^•+^. Antimicrobial activity was also assessed. The obtained minimum inhibitory concentrations (MIC) of these extracts ranged from 12.5 to 50 µg·mL^−1^ against *Micrococcus luteus*, *Listeria ivanovii*, *Staphylococcus aureus*, *Salmonella enterica*, *Escherichia coli*, *Pseudomonas aeruginosa*, *Aspergillus niger*, and *Fusarium oxysporum*, and from 25 to 50 µg·mL^−1^ against *Candida albicans*. Therefore, these extracts can be considered as a potential source of biological active compounds.

## 1. Introduction

Plants have been used in traditional medicine for millennia. Medicinal plants are a source of substances with varied biological and pharmacological activities [1]. The Crassulaceae family comprises 33 genera and 1500 species. *Aeonium arboreum* is the main species found in North Africa, especially in Tunisia [2]. There are two varieties of *Aeonium arboreum*; *Aeonium arboreum* var. *atropurpureum* and var *albovariegatum*. The leaves of the first one are more robust than the browner, more fragile leaves of the second, which also had a more developed epicuticular wax layer, an adaptation to life in arid conditions.

The chemical composition of species in the *Aeonium* genus has only been partially described. Using the GC–MS analysis, Stevens et al. have identified the presence of quercetin derivative in the leaves extract of *A.* spp., including *A. arboreum* [3]. This researcher has reported always the identification of tannins, terpenoids, and flavonoids (such as methyl ethers of kaempferol, 6-hydroxykaempferol, quercetin, myricetin, and scutellarein) in the *Aeonium* species. The combination of kaempferol 3,7-dimethyl ether and quercetin 3,7-dimethyl ether is characteristic of *Aeonium* species, only myricetin methyl ethers are found in *A. goochia* and *A. petrothamnium*.

*Aeonium arboreum* has long been used in the traditional medicinal practices of several countries to treat various ailments. It is used as a: diuretic, litholytic [4], antipyretic, febrifuge, antihemorrhoidal [5], and anti-inflammatory [6]. Leaves can be used in fresh or dried form. Infused, they are used to treat heart problems, liver diseases, and bronchial problems. As part of a poultice, they are used to treat headaches and tooth abscesses [7]. The *Aeonium* hydroethanolic extract of *A*. *arboreum* leaves have a powerful antihypertensive effect in rats at a dose of 400 mg.kg^−1^ [8]. Most of the previous studies of these plants were focused on phenolic compounds while other bioactive components and their biological effects have not yet been fully investigated, e.g., antioxidant and antimicrobial activities. Rather than ascorbic acid, a vitamin found mainly in fruits, many medicinal plants contain antioxidants in the form of polyphenols and trolox, a water soluble vitamin E analogue [9]. These substances possess high antioxidant potential and are counterparts to oxidative stress. Therefore, this assay has been expanded to *A.* *arboreum* to measure the total antioxidant capacity, including polyphenols, which are regarded as valuable phytomedicines.

In this study, we analyzed the phytochemical characteristics and chemical composition of EtOAc, hydroethanolic, and *n*-BuOH leaf extracts from *A*. *arboreum*. The in vitro antioxidant and antimicrobial activities of the three extracts were also evaluated. It has been demonstrated that more than one method is necessary to elucidate the antioxidant capacity of samples because these assays differ in the principal and experimental conditions [10,11]. Thus, we used four different complementary antioxidant assays (FRAP, TAC, DPPH, and ABTS) and compared to that of commercial antioxidants such as vitamin C and Trolox. To our knowledge, this is the first study to investigate on the chemical composition and the antioxidant and antimicrobial activities of Tunisian *A*. *arboreum*.

## 2. Results and Discussion

### 2.1. Total Phenolics, Flavonoids, and Condensed Tannins Contents of Various Extracts from A. arboreum Leaves

For all extracts, three families of compounds were identified: total phenolic content (TPC), total flavonoid content (TFC), and condensed tannins (CTs). Table 1 summarizes the chemical composition of *A. arboreum* leaf extracts. The hydroethanolic extracts were richer in phenolics (100.988 mg GAE/g) and flavonoids (94.811 mg QE/g) (*p* < 0.05) followed by the EtOAc and *n*-BuOH extracts, but had the lowest condensed tannins (9.320 mg CE/g).

In a previous study, comparing three plants belonging to the Crassulaceae family [12] flavonoids content did not exceed 36.4 mg QE/g of extract. This result indicates the richness of *A. arboreum* in phenols, mainly flavonoids.

### 2.2. Phytochemical Constituents

**Figure 1:**
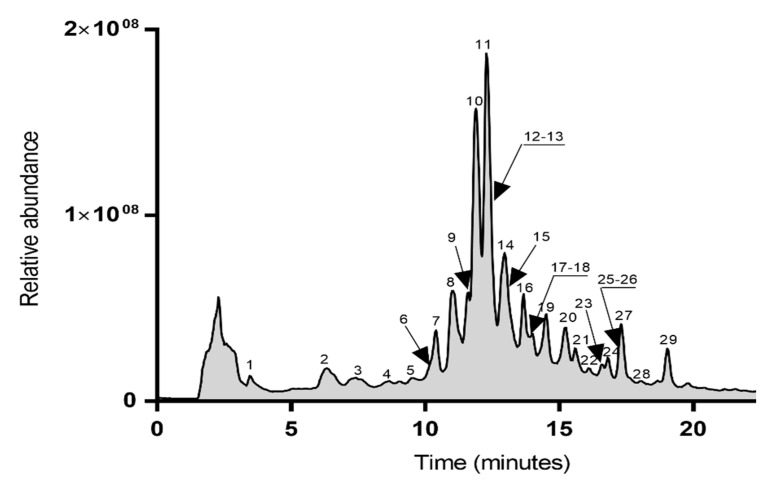
Total ion chromatogram of LC/ESI(−)/MS of the *A. arboreum* leaf (EtOH–water, 70/30, *v/v*) extract.

The myricetin and quercetin glycosides present in the hydroethanolic (EtOH–water, 70/30, *v/v*) extract of *A*. *arboreum* were described and then characterized using LC and LC–MS/MS. The total ion mass chromatogram profile of this extract is shown in Figure 1.

To identify the glycosides attached to aglycones, we measured the loss of sugar units (Table S1). The mass loss of monosaccharides and of disaccharides was deduced using mass spectrometry.

The LC-MS/MS analysis of *A. arboreum* leaf extract (EtOH–water, 70/30, *v/v*) revealed thirty four compounds (Table 2).

Peaks 1a, 1b, 2, and 3 were attributed to isocitric acid, malic acid, 1,1,2-ethandicarboxylic acid, and 1,1,2,3-propantetracarboxylic acid. These compounds were characterized by their mass spectra and by their UV spectra between 257 and 263 nm.

Peaks 4–29 are flavonoids, as characterized by their UV spectra, the two major absorption peaks between 250 and 370 nm are characteristic of myricetin and quercetin glycosides. In order to obtain structural information of the dominant flavonol glycoside compounds, a preliminary MS full scan mode analysis led to the identification of predominant *m/z* ratios and was followed by MS/MS full scan acquisition. The first order spectra of flavonoid glycosides produced deprotonated molecular ions [M-H]^-^. These were used as precursors in the LC–MS/MS analysis, producing fragment ions corresponding to deprotonated Y_0-_ aglycones generated by the loss of sugar units. Thus, the second order spectra MS^2^ allowed us to confirm the fragments of flavonol aglycones. The resulting molecular ions and fragments were observed for aglycones at *m/z* 301, 317, 332, and 333, respectively, for quercetin and its derivatives [13].

**Figure 2:**
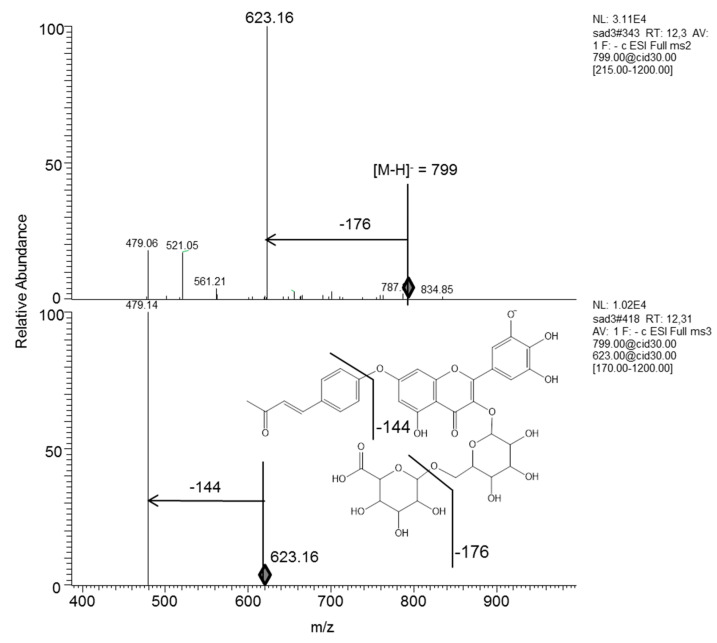
ESI-MS^n^ mass spectra of [M-H]^−^ generated from compound 11 (*m/z* 799): myricetin-3-*O*-glucoside glucuronic acid-7-O-*p*-hydroxybenzalacetone.

- Peak 1 (T_R_ = 3.44 min) was identified as a mixture of isocitric acid (IA), *m/z* 191, and malic acid (MA) *m/z* 133. Mass spectrum of IA showed an ion at *m/z* 191 [IA-H]^−^ and a fragment at *m/z* 155, which was the result of an additional structural rearrangement from a two dehydration, which was then decarboxylated (−44 Da) in an ultimate ion at *m/z* 111 [IA-H-2H_2_O-CO_2_]^−^. Therefore, ions at *m/z* 155 and 111 were characteristic of IA conjugates [15]. Similarly, for MA, the molecular ion at *m/z* 133 was dehydrated (−18 Da) to give a single and characteristic ion at *m/z* 115 [MA-H-H_2_O]^−^.
- Peak 2 (T_R_ = 6.32 min) was identified as 1,1,2-ethanetricarboxylic acid (ETA), giving a molecular ion [M-H]^−^ at *m/z* 161 and two fragment ions at *m/z* 143 and *m/z* 115 [15]. Dehydration and cyclization of this parent ion resulted in a characteristic ion at *m/z* 143 [ETA-H-H_2_O]^−^, which then underwent the loss of CO (−28 Da) to form another single product ion at *m/z* 115 [ETA-H-H_2_O-CO]^−^.
- Peak 3 was identified as propane-1,1,2,3-tetracarboxylic acid, giving a molecular ion at *m/z* 219, undergoing the loss of HCOOH to give an ion at *m/z* 173. This product ion was then dehydrated (−18 Da) to produce an ion at *m/z* 155, which underwent decarboxylation (−44 Da) to produce an ion at *m/z* 111.
- Peaks 4, 9, 11, 13, 15, 16, 20a, 22, 25, 27, 28, and 29 showed myricetin 3-*O*-glucoside as aglycone monosaccharide for these ionic products. For example, the peak 4 at T_R_ = 8.72 min corresponds to a molecular ion at *m/z* 479 [M-H]^−^ with a fragment at *m/z* 317 resulting from the loss of a glucose group (162) indicates myricetin-3-*O*-glucoside. The peak 9 at T_R_ = 11.6 min corresponding to a molecular ion at *m/z* 655 was assumed to be myricetin-3-*O*-glucoside-5-*O*-glucuronic acid by its MS^2^ fragment ion at *m/z* 479, corresponding to the loss of glucuronic acid. The major peak 11 at T_R_ = 12.29 min indicates a parent ion [M-H]^−^ at *m/z* 799 with fragments at *m/z* 623, 479, and 317 (Figure 2). The pseudo molecular ion [M-H-glucuronic acid]^−^ at *m/z* 623 was identified as myricetin-3-*O*-glucoside-5-*O*-*p*-hydroxybenzal-acetone according to the fragmentation scheme proposed by Han et al. [32]. The loss of *p*-hydroxybenzalacetone gives a daughter ion at *m/z* 479 identified as myricetin-3-*O*-glycoside-*O*-glucuronic acid 5-*O*-*p*-hydroxybenzalacetone. The Peak 13 at T_R_ = 12.98 min showed a molecular ion at *m/z* 479 and MS^2^ ion at *m/z* 316, indicating myricetin-hexoside. Peak 15 at 13.25 min was also assigned to myricetin. The ion at *m/z* 915 [M-H]^−^ and a fragment ion of 739 validated the loss of glucuronic acid. The peak 20a at T_R_ = 15.22 min presented [M-H]^−^ at 801 with its fragments *m/z* 625 and 479, which indicates the presence of myricetin-3-*O*-glucoside. Moreover, the MS^2^ daughter ion at *m/z* 625 indicates glucuronic acid (−176 Da). The difference between these two ions (*m/z* 625 and *m/z* 479) gives *m/z* 146, revealing the rhamnose group. Therefore, the *m/z* 801 compound was identified as myricetin-3-*O*-glucoside-7-*O*-rhamnopyranoside-*O*-glucuronic acid.

- Peaks 7, 8, 14, and 20b at T_R_ = 10.38, 11, 13, and 15.22 min respectively lead to observation of a common ion at *m/z* 495, which could be attributed to 3,3′,4′,5,7-pentahydroxy-6-methoxyflavanonol-3-*O*-glucoside. Analysis of the MS^2^ spectra of peak 7 showed intense ion *m/z* at 332, corresponding to the loss of glucoside. In contrast, peak 8 consisted of two compounds, which could be characterized with two different mass spectra: Firstly, peak 8a matched with a molecular ion at *m/z* 787 [M-H]^−^ with MS^2^ fragment ions at *m/z*, 611 and 495, arising from the loss of glucuronic acid (*m/z* 176) and the loss of a dirhamnoside (*m/z* 292) respectively. Then, we observed in peak 8b, the *m/z* 671 with a MS^2^ fragment at *m/z* 495 following the loss of a glucuronic acid.
- Peak 12 at T_R_ = 12.89 min, with a molecular ion at *m/z* 783 and its MS^2^ spectrum, indicates the eupafolin-3,7-di-*O*-rhamnoside 4′-*O*-glucuronic acid according to the fragmentation scheme proposed by Chiung-Sheue Liu et al. [23]. Peaks 17 and at T_R_ = 14.01 and 14.3 min respectively were attributed to three quercetin glucosides with different positions of glucoside. In fact, they revealed the same MS^3^ ion at *m/z* 301 corresponding to quercetin aglycone arising from the loss of glucoside. Analysis of peak 21 at T_R_ = 15.61 min showed characteristic ions at *m/z* 447, attributed to quercetin-7-*O*-rhamnoside. MS^2^ fragment at *m/z* 301 indicates the loss of a rhamnoside unit.
- Peaks 19a and 23 at T_R_ = 14.47 and 16.59 min respectively could be attributed to patuletin 3-*O*-rhamnopyranosyl-7-*O*-rhamnopyranoside-4′-*O*-glucuronic acid and patuletin-7-*O*-glucoside due to the molecular ions (*m/z* 827 and *m/z* 493 respectively) and their MS^2^ and MS^3^ spectra.
- Peak 20b at T_R_ = 15.22 min, leads to the observation of a molecular ion at *m/z* 817 [M-H]^−^ with fragment ions at *m/z* 641 and 495 indicating the successive losses of glucuronic acid and rhamnose. Therefore, this compound was identified as 3, 3′,4′,5, 7-pentahydroxy-6-methoxyflavonol-3-*O*-glucoside-5-*O*-rhamnopyranoside-*O*-glucuronic acid.

### 2.3. Antioxidant Activity of Leaf Extracts In Vitro

**Figure 3:**
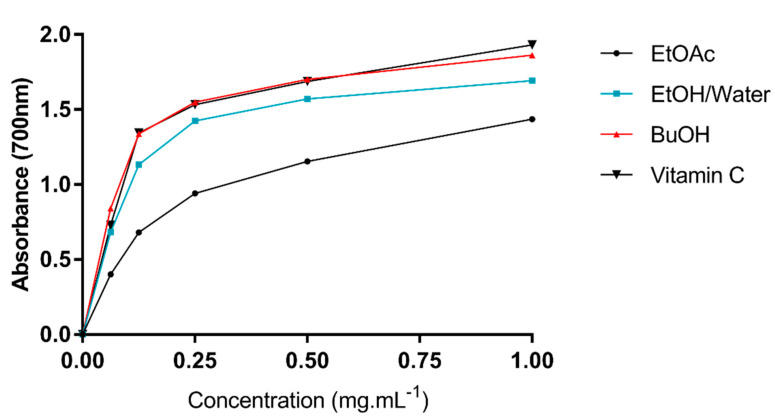
Ferric reducing power of *A. arboreum* leaf extracts compared to vitamin C as a standard (*n* = 3).

Ferric reducing activity power (FRAP). This method is based on the reduction of the Fe^3+^ TPTZ complex (colorless complex) to Fe^2+^-tripyridyltriazine (blue complex) by electron-donating antioxidants at low pH. The reducing power of extracts and vitamin C were also determined (Figure 3). In this test, all extracts presented dose-dependent activity whose results are lower than that of vitamin C as a standard. There is a positive correlation between the reducing power of *A. arboreum* extracts and concentration increase (Figure 3). *n*-BuOH extract had the highest reducing power (*p* < 0.05), followed by hydroethanolic and EtOAc extracts.

The reducing power of extracts depends on the presence of reducing agents giving a hydrogen atom. It has been reported that reductones, responding to various precursors of peroxides, prevent their generation [28]. The differences in solvent polarities and thus a different extract ability of the antioxidant components may indicate the potential antioxidant capacity of *A. arboreum* extracts. The hydroalcoholic and EtOAc extracts have the strongest antioxidant effects, related to their high levels of phenolic and flavonoid contents (Table 3). Thus, *A. arboreum* extracts could be used as natural antioxidant agents.

**Figure 4:**
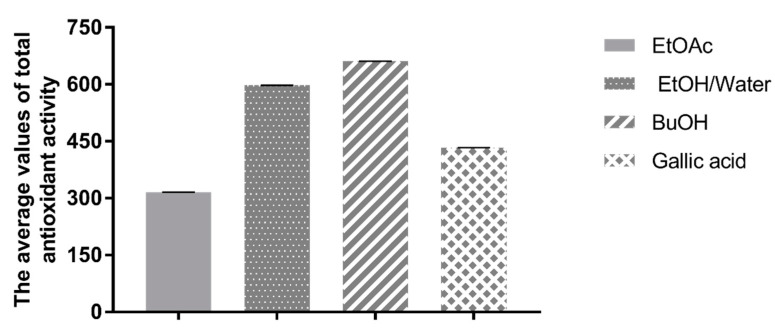
Total antioxidant capacity of *A. arboreum* leaf extracts compared to gallic acid as a standard.

Total antioxidant capacity (TAC): The TAC of the extracts was measured using the phosphomolybdenum method to assess their broader antioxidant potential [30] (Table 3, Figure 4). The antioxidant compound reduces Mo(VI) to Mo(V) by forming a green phosphomolybdenum complex (V) with maximal absorption at 695 nm. *n*-BuOH and EtOH/water extracts had the highest antioxidant capacities (661.429 mg gallic acid equivalents (GAE)/g extract and 597.934 mg GAE/g extract respectively), compared to the gallic acid (433.198 mg GAE/g extract) and followed by EtOAc extract, which may be related to its high levels of total phenolics (TP) and total flavonoids (TF) contents.

**Figure 5:**
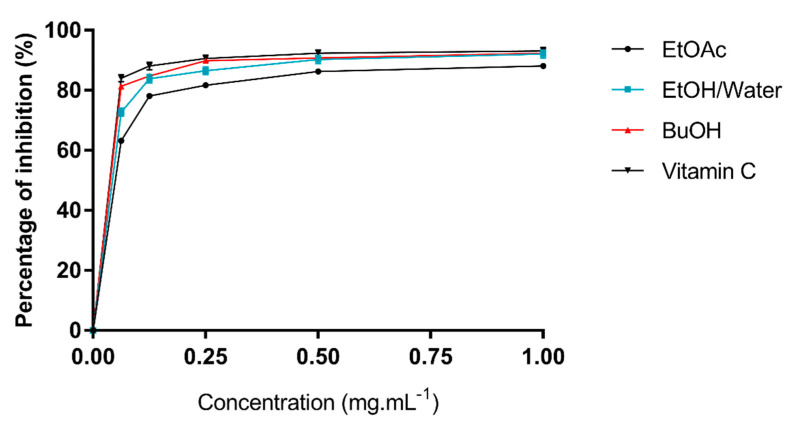
Scavenging activity of DPPH^•^ radical assay of *A. arboreum* leaf extracts at different concentrations compared to vitamin C as a standard.

DPPH^•^ and ABTS^•+^ scavenging activity. Various phenols and flavonoids were evaluated for their DPPH^•^ and ABTS^•+^ scavenging activity and are present in many products of plant origin, for example *Sedum sempervivoides*, which belongs to the Crassulaceae family. The methanolic extract of this plant has shown (Figure 5) a low DPPH^•^ scavenging activity (IC_50_ = 0.082 mg·mL^−1^) [30] compared to the hydroethanolic extract of *A. arboreum* (IC_50_ = 0.037 mg·mL^−1^). The *n*-BuOH extract of *A. arboreum* has a powerful scavenging activity of DPPH^•^ (IC_50_= 0.031 mg·mL^−1^) compared to that of vitamin C (IC_50_= 0.025 mg·mL^−1^). Our results suggest that hydroxyl groups of the three extracts can act as electron donors, converting free radicals into more stable substances by trapping radicals.

In this method, the peroxidation reaction is triggered by a reactive radical extracting an electron from a non-radical. The radical DPPH^•^ is transformed to a nonradical DPPHH. In the presence of flavonoids in the polar extracts, an electron can be given from hydrogen from the phenolic group, and the new radical formed is more stable than before [31]. Our LC/MS analysis indicated the presence of active -OH groups in this extract. In this regard, the antioxidant capacity of flavonoid compounds depends on the number of hydroxyl groups [29].

**Figure 6:**
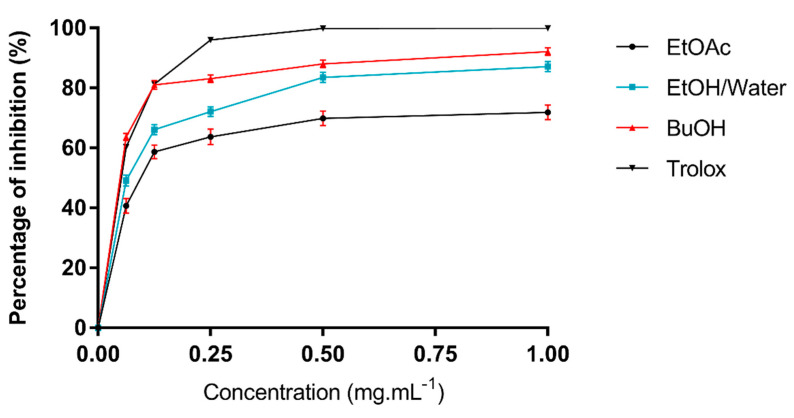
Scavenging activity of *A*. *arboreum* leaf extracts on ABTS^•+^ radical compared to the Trolox standard.

Scavenging capacity by ABTS^•+^ is shown in Table 3. The results of the assay were strongly and positively correlated to those of the DPPH^•^ assay (Table 4). The deduced IC_50_ values show that the EtOH–water and *n*-BuOH extracts (IC_50_= 0.0625 mg·mL^−1^ and IC_50_= 0.048 mg·mL^−1^ respectively) had strong scavenging activity whereas those of ethyl acetate had moderate scavenging activity (IC_50_= 0.09 mg·mL^−1^) compared to Trolox (IC_50_= 0.051 mg·mL^−1^) (Figure 6). This could be related to the nature of the compounds. In general, DPPH^•^ and ABTS^•+^ scavenging activity assays measured antioxidant reductive capacity. We can conclude that flavonoids are able to scavenge free radicals and can interact with reactive oxygen species [33].

### 2.4. Correlations

A significant correlation (*p* < 0.01) was found between ABTS^•+^ and FRAP (r^2^ = 0.983), and a lower correlation between TFC and DPPH^•^ (r^2^ = 0.786) (Table 5). TFC and FRAP were found to be weakly positively correlated (r^2^ = 0.696, *p* < 0.05). TPC and TFC were found to be highly correlated (r^2^ = 0.962). There was correlation between FRAP and CAT (r^2^ = 0.798), TPC and ABTS^•+^ (r^2^ = 0.837), and TPC and FRAP (r^2^ = 0.858) at *p* < 0.01. The correlation between CTs and DPPH^•^ (r^2^ = 0.583) was, at *p* < 0.05, relatively lower (Table 5).

### 2.5. Antimicrobial Activity of A. arboreum

The antimicrobial activity of the different extracts (EtOAc, EtOH/water and *n*-BuOH) was investigated against nine microorganisms (six bacteria (three Gram-positive and three Gram-negative), two fungi, and one yeast strain) and two controls (ampicillin for bacteria and amphotericin B for fungi and yeast), and assessed quantitatively by their minimum inhibitory concentration (MIC), minimum bactericidal concentration (MBC), and minimum fungicidal concentration (MFC) values.

The results, summarized in Table 5, showed that the three extracts had a strong antibacterial effect, producing zones of inhibition with diameters from 11 to 20 mm, while the standard antimicrobial agent ampicillin produced zones of inhibition ranging from 10 to 27 mm. The hydroalcoholic extract had the strongest antibacterial activity against *Micrococcus luteus* (20 ± 0.8 mm), *Salmonella enterica* (14 ± 0.3 mm), *Pseudomonas aeruginosa* (18 ± 0.3mm), *Fusarium oxysporum* (22 ± 0.3 mm), and *Candida albicans* (20 ± 0.3 mm), about two times higher than the positive control. The *n*-BuOH extract showed the highest antibacterial activity against *Salmonella enterica* (15 ± 0.3 mm) and *Pseudomonas aeruginosa* (16 ± 0.3 mm), higher than ampicillin (10 ± 0.3 mm and 11 ± 0.8 mm, respectively). The ethyl acetate extract showed significantly higher antibacterial activity against *Salmonella enterica* (15 ± 0.3mm) and *Pseudomonas aeruginosa* (20 ± 0.3 mm) than the positive control.

The composition of the extracts greatly influenced their antimicrobial properties. The higher resistance of Gram (−) bacteria could be explained by the outer membrane covering the cell wall, whose phospholipid coating limits the diffusion of hydrophobic compounds [34]. For Gram (+) bacteria, the absence of this barrier allows direct contact of this extracts with the phospholipids bilayer of the cell membrane, which causes increased permeability to ions and the passage of vital intracellular constituents or an alteration of bacterial enzymatic systems [35]. The results of the MIC, MBC, and MFC (Table 6) values were found to vary with the extraction solvent and were generally in accordance with those recorded for inhibition zones (Table 5). The MBC values ranged between 25 and 50 mg·mL^−1^, whereas the MIC values were similar or half the MBC values.

According to the literature, an extract is bactericidal (B) when the ratio MBC/MIC is lower than 4 and bacteriostatic (b) if MBC/MIC is higher than 4 [34]. Only the hydroethanolic extract had a bactericidal effect with MBC/MIC ratio lower than 4 against *Staphylococcus aureus* whereas the ethyl acetate and *n*-butanolic extracts showed bacteriostatic effects. However, for the fungicidal degree of extracts, as described by the MFC/MIC ratios, the results showed that the ethyl acetate and hydroethanolic extracts possess a fungistatic degree for *Fusarium oxysporum* and *Candida albicans*, but the *n*-butanolic extract was inactive. The biological activity of this extract is linked to its chemical composition and to the functional groups of the main compounds such as flavonoids in this work (myricetin, quercetin, and 3,3′,4′,5,7-pentahydroxy-6-methoxyflavanonol).

## 3. Materials and Methods

### 3.1. Extraction

Fresh *A. arboreum* leaves were collected on 26 March 2018 from Sfax, central Tunisia (34°44′ N and 10°43′ E). The plant was identified by Pr. Mohamed Chaieb, Biology Department, Faculty of Sciences of Sfax, and a voucher specimen (LCO 143) was deposited at the herbarium of the Laboratory of Organic Chemistry (LR17-ES08), Natural Substances Team, Faculty of Sciences, University of Sfax. Leaves (1.2 kg) were macerated with EtOH/water (70/30, *v/v*) at room temperature three times at 24 h intervals and filtered to obtain the hydroethanolic extract, which was concentrated under reduced pressure (40 °C) to remove ethanol. The 500 mL remaining aqueous phase was lyophilized and then 1.15 ppm of hydroalcoholic extract were obtained and the remaining aqueous phase was then successively extracted by liquid/liquid extraction using solvents with increasing polarity; *n*-hexane, ethyl acetate, and *n*-butanol. After solvent evaporation, the obtained extracts, 2.425 ppm, 2.635 ppm, and 3.680 ppm respectively, were stored at 4 °C prior to analysis [9].

### 3.2. Determination of Phenolic, Flavonoid, and Condensed Tannins Contents

- Total phenol content (TPC) was determined using the spectrophotometric/colorimetric method described by Mhalla et al. (2017) [34]. This assay quantifies the total hydroxyl groups in the three extracts. A total of 0.5 mL of the Folin–Ciocalteu reagent [36] was added to a solution containing 1 mL of the extract (EtOAc, hydroethanolic, or *n*-BuOH) with a known concentration (1 mg·mL^−1^) and 3 mL of distilled water. A total of 0.5 mL of 2% aqueous sodium carbonate (Na_2_CO_3_) was added after 5 min. The mixture was then incubated at 25 °C for 90 min and absorbance measured at 760 nm. The assay was performed in triplicate for each extract. The TPC was calculated by a standard gallic acid graph, with TPC expressed in milligrams of gallic acid equivalent per gram of dry weight of extract.
- Total flavonoid content (TFC) was determined using the method described by Akrout et al. [37] and Mhalla et al. [34]. The method is based on the formation of a very stable complex, between aluminum chloride and the oxygen atoms present on carbons 4 and 5 of the flavonoids, which has maximum absorbance at 430 nm. Quercetin was used to make the calibration curve. One milliliter of sample (1 mg·mL^−1^) was mixed with 1 mL of 2% aluminum trichloride (AlCl_3_) MeOH solution. After incubation at room temperature for 15 min, the absorbance of the mixture was measured at 430 nm with a Shimadzu UV-mini 1240 UV/VIS spectrophotometer with TFC expressed in milligrams of quercetin equivalent (QE) per gram of extract.
- Condensed tannins (CTs) content was determined using the method described by Mhalla et al. [34]. In this method vanillin and HCl react with the flavonoid group and form red complexes [9,36] or anthocyanidols [38,39]. A total of 50 μL of each extract was added to 1500 μL of 4% vanillin/methanol solution and mixed vigorously before 750 μL of concentrated hydrochloric acid were added. The mixture was left to react at ambient temperature for 20 min. Absorbance was measured at 550 nm against a blank. Different concentrations (between 0 and 1000 µg·mL^−1^) prepared from a stock solution of catechin allowed the calibration curve to be drawn.

### 3.3. RP-HPLC Fractionation and LC–MS Analysis

Fractionation of the hydroethanolic extract was carried out using a Finnigan Spectra SYSTEM HPLC equipped with a DAD-UV 6000LP detector and a Phenomenex C18 reversed phase Luna column (5 µm, 150 mm × 4.60 mm) and controlled by ChromQuest 5.0 software. The flow of the mobile phase was 1 mL.min^−1^ and the solvent system was 0.1% formic acid in water (solvent A) and 80% aqueous acetonitrile and 0.1% acidic formic (solvent B). The elution was carried out with a linear gradient of 10–100% of solvent B for 40 min and a UV detection of this extract was carried out between 200 and 600 nm. The fractions were collected, vacuum-dried and directly analyzed by mass spectrometry.

Ten microliters of each fraction were analyzed with a Surveyor HPLC equipped with a Phenomenex C18 reversed-phase column Luna (5 µm, 150 mm × 2.1 mm) at a flow rate of 200 µL.min^−1^. The system was coupled to an ion trap LCQ Advantage mass spectrometer (Thermo Finnigan, Courtaboeuf, France) fitted with an electrospray ionization source in the negative mode. Spray voltage was at 4.5 kV, the capillary temperature set at 300 °C, and sheath and auxiliary gas set at 50 and 5 psi respectively. The acquisition range was from 100 to 2000 *m/z*. The method combined full scans and MS/MS experiments using a collision energy ranging from 10 to 35 eV, depending on the molecular mass of compounds.

The area value of each peak was integrated using Xcalibur software (version 4.0, ThermoFisher Scientific, Courtaboeuf, France). The relative peak area indicates the contribution of each compound to all identified in the extract, providing a measure of relative abundance.

### 3.4. Biochemical Assays

Ferric-reducing antioxidant power assay: The ferric reducing power of *A. arboreum* leaf extracts was measured using the method described by Ben Younes et al. [35]. Extracts (1 mg) were dissolved in 1 mL of EtOH and mixed with 2.5 mL of 0.2 mol.L^−1^ sodium phosphate buffer (pH = 6.6) and 2.5 mL of 1% potassium ferricyanide (K_3_Fe (CN)_6_), then incubated in a water bath at 50 °C for 20 min. Then, 2.5 mL of 10% trichloroacetic acid was added to the mixture, which was centrifuged for 10 min. The supernatant (2.5 mL) was then mixed with 2.5 mL of distilled water and 0.5 mL of 0.1% ferric chloride solution. The absorbance of the mixture was measured at 700 nm. Absorbance increases with reducing power. A standard curve was created using four various concentrations of vitamin C: 0.0625, 0.25, 0.5, and 1 mg·mL^−1^. The assays were carried out in triplicate.

Total antioxidant capacity: The antioxidant activities of the extracts were evaluated by the method of complex formation with phosphomolybdenum [35]. This method is based on the transfer of electrons and the reduction of ammonium molybdate. During the reaction, a greenish ammonium phosphate/molybdate complex was formed and detected at 695 nm.

DPPH radical scavenging activity: The antiradical activity of the extracts was evaluated with a colorimetric method using the radical 2,2-diphenyl−1-picrylhydrazyl (DPPH), as described by Ben Younes et al. [35]. The DPPH radical had a deep violet color (515 nm) in solution and it became colorless when neutralized, i.e., after saturation of its electronic layers. UV spectrometry was used to measure the decrease in absorption at 515 nm. Vitamin C was used as the standard for concentrations 1 mg·mL^−1^ of plant extract and all tests were carried out in triplicate. The percentage of inhibition (PI%) of DPPH radicals was calculated as:

DPPH radicals PI (%) = [(DO_b_ − DO_a_)/DO_b_] × 100

where DO_b_ refers to the absorbance of control (without extract) and DO_a_ to the absorbance of sample (with extract).

Antioxidant activity by the ABTS^•+^ method: The 2,2-hydrazine-bis (3-ethyl-benzothiazoline-6-sulfonic acid) diamine (ABTS) radical scavenging activity was determined using the method described by Re et al. [40]. This test is based on the ability of an antioxidant to stabilize the cationic free blue-green colored radical ABTS^•+^ generated in the presence of persulphate ions by transforming it into colorless ABTS. The reaction was followed by the measurement of the optical density at the wavelength 734 nm. A total of 7 mmol.L^−1^ of ABTS^•+^ and 2.45 mmol.L^-1^ of K_2_S_2_O_8_ were mixed and then incubated for 12–16 h in a darkroom at 4 °C, in order to prepare ABTS^•+^ free radicals. The mixture was dissolved in distilled water to create a stable color. Subsequently, the protocol described by Ben Salem et al. [41] was used

The antioxidant capacity of the extracts was expressed quantitatively in mmol of Trolox equivalents (TE) (mmol TE/g of dry extract).

ABTS^•+^ radicals PI (%) = [(DO_b_ − DO_a_)/DO_b_ ] × 100

where DO_b_ refers to the absorbance of the control (without extract) and DO_a_ to the absorbance of the sample (with extract).

### 3.5. In Vitro Evaluation of Antimicrobial Activity

Microbial strains and conditions: The extracts of this plant were evaluated against a panel of microorganisms comprising nine bacterial strains (Gram positive (*Staphylococcus aureus* (ATCC 6538), *Micrococcus luteus* (LB 14110), and *Listeria ivanovii* (BUG 496)), Gram negative (*Salmonella enterica* (CIP 8039), *Escherichia coli* (ATCC 8739), and *Pseudomonas aeruginosa* (ATCC 9027)), fungal (*Fusarium oxysporum* (GCA_003615085) and *Aspergillus niger* (CBS 513.88)), and a yeast (*Candida albicans* (ATCC 90028)). The wells were then filled with 60 µL of the extract at 20 mg·mL^−1^ in 5% DMSO (5% DMSO in water was used as a negative control). All strains tested were obtained from the Department of Microbiology, Faculty of Sciences, University of Sfax (Tunisia). The fungal and yeast strains were grown for 48 h at 30 °C in Sabouraud chloramphenicol (SCA) agar, while the bacteria strains were grown for 24 h at 37 °C in Mueller–Hinton agar (MHA).

Disk diffusion method: The disk diffusion method was used according to Mhalla et al. [34] to determine the antibacterial activity of the extracts. For this evaluation, culture suspension (150 µL) stumps (10^6^ colony forming units (CFU mL^−1^)) of the bacteria studied were spread over the surface of the solid MHA support plates. Sterilized filter paper discs (5 mm in diameter) were soaked with 10 μL of extract (2.5 mg mL^−1^) and placed on the inoculated plates. The antibacterial and antifungal activities were evaluated by measuring the diameters of the inhibition zones of the tested microorganisms and compared to specific antibiotics. Ampicillin (10 µg per disc) and amphotericin B (20 µg per disc) were used as positive controls against bacteria, fungi, and yeasts, respectively. Tests were reproduced in triplicate. The zone of inhibition refers to the degree of sensitivity of bacteria or fungi to an extract according to the following criteria [42]: D < 6 mm: resistant (no activity), 6 mm < D < 13 mm: moderate activity, and D > 13 mm: strong antimicrobial activity.

Microdilution method: To determine the minimum inhibitory concentration (MIC) and minimum bactericidal concentration (MBC), we used the method of microdilution in broth described by Bassole et al. [43]. After the strains culture, inocula were suspended in MHB to provide a final density of 10^6^ CFU mL^−1^. Then, in a 96-well microplate, the extracts dissolved in sterilized distilled water were used in two folds dilutions ranging from 2.5 to 0.019 mg·mL^−1^. Ampicillin and amphotericin B were used as standard references against bacteria, fungi, and yeast respectively. These plates were then incubated under normal atmospheric conditions at 37 °C for 24 h for bacteria and at 30 °C for 48 h for yeast and fungi according to the work of Boshir et al. [44]. In proportion to the ratios, the effect is bactericidal (fungicidal) or bacteriostatic (fungistatic):

- 1 < MBC/MIC < 4: bactericidal action;
- 4 < MBC/MIC < 16: bacteriostatic action;
- 1 < MFC/MIC < 4: fungicidal action;
- 4 < MFC/MIC < 16: fungistatic action.

### 3.6. Statistical Analysis

Statistical significance of differences was evaluated using a one-way ANOVA, followed by Tukey’s post hoc test for multiple comparisons with *p* = 0.05 and correlation coefficients (r). These analyses were carried out using the Statistical Product and Service Solutions program (SPSS) version 20.

## 4. Conclusions

This paper is the first to highlight the antioxidant and antibacterial properties of *A. arboreum* hydroethanolic extract and its high total phenolic and flavonoid contents. Twenty-one out of thirty-four phenolic compounds in this extract were quantified using an adapted LC-ESI-MS/MS method and identified by comparison of their mass spectra with the literature. These phenolic compounds allowed the samples to present a significant antioxidant activity in four different methods: TAC, FRAP, ABTS, and DPPH. The highest activity was detected in *n*-BuOH extract, followed by the EtOH/water and EtOAc extracts, respectively. Vitamin C, vitamin E, gallic acid, tannins, and total phenolics contents correlated positively with the antioxidant index. These results suggest that the antioxidant activities of this plant are attributed to the chemical components present the phenolic acid and flavonoids. Additionally, the hydroethanolic extract demonstrates promising antimicrobial activity against food-borne pathogens and spoilage bacteria *Micrococcus luteus*, *Staphylococcus aureus,* and *Pseudomonas aeruginosa* known for their resistance to antibiotics. Additionally, this extract displayed a significant antifungal effect against *Fusarium oxysporum*, but not against *Aspergillus niger*. All this suggest a potential use for the hydroethanolic extract as a natural preservative in the food and/or pharmaceutical industries.

Our results showed that the antioxidant activity was mostly dependent on the solvent used in the extraction procedure. However, further research is required to isolate new bioactive components in the extracts, to identify those producing the activities observed and to evaluate their in vivo biological capacities.

## Figures and Tables

**Table 1:** Chemical composition of *A. arboreum* leaf extracts.

Extracts	TPC (mg GAE/g)	TFC (mg QE/g)	CT(mg CE/g)
EtOAc	67.654 ± 0.034 ^b^	60.382 ± 0.000 ^b^	18.640 ± 0.000 ^b^
EtOH/Water (70-30, *v/v*)	100.988 ± 0.046 ^a^	94.811 ± 0.001 ^a^	9.320 ± 0.000 ^c^
*n*-BuOH	64.938 ± 0.002 ^c^	38.136 ± 0.000 ^c^	34.950 ± 0.000 ^a^

Values (mean ± SD.; *n* = 3) in the same column followed by different letter(s) are significantly different (*p* < 0.05) according to Duncan’s multiple range test. GAE: gallic acid equivalent, QE: quercetin equivalent, CE: catechin equivalent. a: high content, b: medium content, and c: low content.

**Table 2:** Identification of compounds from *A. arboreum* leaf (EtOH–water, 70/30, *v/v*) extract by LC–MS/MS (negative mode). The relative peak area indicates the contribution of each compound to all the identified compounds in the extract, providing a measure of relative abundance.

Compound	T_R_ (min)	UV (nm)	Relative Abundance (%)	[M-H]^−^ *m/z*	LC/ESI-MS^2^ *m/z (%)*	MS^3^	MS^4^	Attribution	Ref
**1a**	3.44	257	0.656	191	111,173, 155			Isocitric acid	[14]
**1b**	3.44	257		133	115 (100)			Malic acid	[15]
**2**	6.32		0.943	161	115, 143 (100)			1,1,2-Ethanetricarboxylic acid	[15]
**3**	7.49	263	1.978	219	111, 155, 173 (100)			Propane-1,1,2,3-tetracarboxylic acid	[14]
**4**	8.72	274–304	0.314	479	271, 299 (100), 316, 317			Myricetin-hexoside	[16,17]
**5**	9.48	285	0.183	597	480, 551 (100)			Unidentified	
**6**	10.2	281	0.539	447	401 (100)	161,131, 269 (100)		Unidentified	
**7**	10.38	275–342	1.489	495	287, 332 (100), 333, 479			3,3′,4′,5,7-pentahydroxy-6-methoxyflavanonol-3-*O*-glucoside	[18]
**8a**	11	261–363	0.327	787	495, 611(100), 671			3,3′,4′,5,7-pentahydroxy-6-methoxyflavanonol-3-*O*-glucosyl -5,4′-*O*-dirhamnoside	[18]
**8b**	11	261–363	0.270	671	495 (100)	265, 271, 304, 333 (100),		3,3′,4′,5,7-pentahydroxy-6-methoxyflavanonol-3-*O*-glucoside-5-*O*-glucuronic acid	[18]
**9**	11.6	260–359	5.831	655	479 (100)			Myricetin-3-*O*-glucoside-7-*O*-glucuronic acid	[19]
**10**	11.87	261–363	29.001	815	495, 537, 577, 639 (100)	495(100), 537, 577	287, 332 (100), 494	Unidentified	
**11**	12.29	261–359	33.961	799	479, 521, 561, 623 (100),	317, 479(100), 521,561	255, 317 (100)	Myricetin-3-*O*-glucoside glucuronic acid -7-*O-* *p*-hydroxybenzalacetone	[20,21]
**12**	12.89	267–354	3.247	783	463, 505, 545, 607 (100), 639			Eupafolin-3-7 di-*O*-rhamnopyranoside-4′-*O*-glucuronic acid	[22,23]
**13**	12.98	267–354	4.864	479	316 (100), 317, 461			Myricetin-hexoside	[16,23,24]
**14**	13	270	1.645	843	495, 537, 577, 639 (100), 699, 741	315, 495 (100), 537, 577		Unidentified	
**15**	13.25	261–359	0.213	915	479, 623, 739, 799 (100)			Myricetin-3-*O*-glucoside di-*O*-rhamnopyranoside-7-*O*-*p*-hydroxybenzalacetone	[21,23]
**16a**	13.64	271–357	2.187	827	479, 521, 561, 623 (100), 683	317, 479 (100)		Unidentified	
**16b**	13.64	265–348	0.792	597	387, 417, 447 (100), 459			Unidentified	
**17**	14.01	265–348	0.752	463	301 (100), 317			Quercetin-*O*-glucoside	[13,25]
**18**	14.3	267–353	0.265	463	301 (100), 300			Quercetin-*O*-glucoside	[13]
**19a**	14.47	272–333	2.135	827	479, 521, 651 (100)			Patuletin-3-*O*-(4′′-*O*-acetyl-rhamno pyranosyl)-7-*O*-(2′′′-*O*-acetyl-α-L rhamnopyranoside)-4′-*O*-glucuronic acid	[26]
**19b**	14.47	272–333	1.509	843	495, 667 (100)	332, 495 (100), 537, 577	195, 332(100)	Unidentified	
**20a**	15.22	271–365	0.938	801	479, 625 (100)			Myricetin-3-*O*-glucoside-7-*O*-rhamnopyranoside-*O*-glucuronic acid	[16]
**20b**	15.22	271–365	0.938	817	495, 641 (100)			3,3′,4′,5,7-pentahydroxy-6-methoxyflavanonol-3-*O*-glucoside-5-*O*-rhamnopyranosyl-*O*- glucuronic acid	[27]
**21**	15.61	263–348	0.906	447	179, 301 (100)			Quercetin-7-*O*-rhamnoside	[28,29,30]
**22**	16.1	273	0.464	869	421, 479, 623, 637,781 (100),			Unidentified	
**23**	16.59	272–370	0.616	493	317 (100)			Patuletin-7-*O*-glucoside	[23]
**24**	16.83	272–368	0.872	467	421 (100)			Triterpenoid acid 7-oxo-11-H derivatives	[31]
**25**	16.83	272–368	0.872	725	479 (100)			Unidentified	
**26**	17	272–333	0.371	885	495, 537, 577, 639 (100),	495 (100), 537, 577		Unidentified	
**27**	17.32	272	4.536	869	479, 521, 561, 623 (100)	317, 479 (100), 521,561		Unidentified	
**28**	18.61	274	0.207	739	317, 479 (100)	317 (100)		Unidentified	
**29**	19	272	2.239	883	479, 521, 561, 623 (100)	479(100)	317 (100)	Unidentified	

**Table 3:** Total antioxidant capacity (TAC), DPPH^•^, and ABTS^•+^ radical scavenging activities of EtOAc, EtOH/water, and *n*-BuOH extracts.

Extracts/Standard	TAC (mg GAE/g Extract)	DPPH^•^ IC_50_ (mg·mL^−1^)	ABTS^•+^ IC_50_ (mg·mL^−1^)
EtOAc	315.865 ± 0.001 ^c^	0.043 ± 0.009 ^c^	0.09 ± 0.017 ^c^
EtOH/Water (70/30, *v/v*)	597.934 ± 0.002 ^b^	0.037 ± 0.018 ^b^	0.062 ± 0.012 ^b^
*n*-BuOH	661.429 ± 0.001 ^a^	0.031 ± 0.005 ^a^	0.048 ± 0.009 ^a^
Gallic acid	433.198 ± 0.002	--	--
Vitamin C	--	0.025 ± 0.015	--
Trolox	--	--	0.051 ± 0.001

Values are the mean of three experiments ± standard deviation (SD) in the same column followed by different letter(s) are significantly different (*p* < 0.05) according to Duncan’s multiple range test. a: high activity, b: medium activity, c: low activity.

**Table 4:** Pearson’s correlation coefficients (r) for some of the investigated parameters of the three extracts of *A. arboreum* leaves.

	TPC	TFC	CTs	DPPH^•^	ABTS^•+^	FRAP	CAT
**TPC**	1	-	-	-	-	-	-
**TFC**	0.962 **	1	-	-	-	-	-
**CTs**	0.227 ns	−0.008 ns	1	-	-	-	-
**DPPH^•^**	0.906 **	0.786 **	0.583 *	1	-	-	-
**ABTS^•+^**	0.837 **	0.663 *	0.652 *	0.929 **	1	-	-
**FRAP**	0.858 **	0.696 *	0.673 *	0.977 **	0.983 **	1	-
**CAT**	0.628 *	0.435 ns	0.557 ns	0.667 *	0.894 **	0.798 **	1

Pearson’s correlation coefficients using the 95% confidence interval. TPC: total phenolics content, TFC: total flavonoids content, CT: condensed tannins, DPPH^•^: antioxidant capacity based on the DPPH^•^ assay, ABTS^•+^: antioxidant capacity based on the ABTS^•+^ assay, FRAP: antioxidant capacity based on the FRAP assay, TAC: total antioxidant capacity based on the QuantiChrom™ antioxidant assay, ns non-significant, ** *p* < 0.01; * *p* < 0.05 (two-tailed).

**Table 5:** Antimicrobial activity of EtOAc, hydroethanolic, and *n*-BuOH leaf extracts of *A. arboreum* against bacterial, fungal, and yeast strains by the disc method.

Strains	Inhibition Zones Diameter (mm) ^a^			
	Antibiotic/Antifungal	AcOEt Extract	EtOH-Eau (70/30) Extract	*n*-BuOH Extract
Gram (+) bacterial strains ^b^				
*Micrococcus luteus* LB141110	17 ± 0.3	16 ± 0.0	20 ± 0.8	19 ± 0.3
*Listeria ivanovii* BUG 496	22 ± 0.3	17 ± 0.3	11 ± 0.3	16 ± 0.8
*Staphylococcus aureus* ATCC 6538	27 ± 0.8	20 ± 0.3	20 ± 0.8	20 ± 0.3
Gram (−) bacterial strains ^b^				
*Salmonella enterica* CIP 8039	10 ± 0.3	20 ± 0.3	14 ± 0.3	15 ± 0.3
*Escherichia coli* ATCC 8739	22 ± 0.3	16 ± 0.3	13 ± 0.3	16 ± 0.3
*Pseudomonas aeruginosa* ATCC 9027	11 ± 0.8	20 ± 0.3	18 ± 0.3	16 ± 0.3
Fungal strains ^c^				
*Aspergillus niger* GCA_003615085	NA^d^	NA	NA	NA
*Fusarium oxysporum* CBS 513.88	20 ± 0.3	21 ± 0.8	22 ± 0.3	20 ± 0.3
Yeast strain ^c^				
*Candida albicans ATCC* 90028	19 ± 0.3	20 ±0.3	20 ± 0.3	18 ± 0.2

Values are the mean of three experiments ± standard deviation (SD). (a) Diameter of the inhibition zones of extract, including the diameter of a well of 6 mm. (b) Ampicillin was used as a standard antibiotic at a concentration of 10 µg/well. (c) Amphotericin B was used as a standard fungicide and antiyeast at a concentration of 20 µg/well. (d) No activity.

**Table 6:** Determination of the minimum inhibitory concentration (MIC), minimum bactericidal concentration (MBC), and minimum fungicidal concentration (MFC) of *A*. *arboreum* leaf extracts by the microdilution method.

	Extracts	Ampicillin/ Amphotericin B	EtOAc Extract			Inter. ^b^	EtOH–Water (70/30, *v/v*) Extract			Inter. ^b^	*n*-BuOH Extract			Inter. ^b^
Strains		MIC (µg·mL^−1^)	MIC (µg·mL^−1^)	MBC; MFC (µg·mL^−1^)	R ^a^		MIC (µg·mL^−1^)	MBC; MFC (µg·mL^−1^)	R		MIC (µg·mL^−1^)	MBC; MFC (µg·mL^−1^)	R	
		Gram (+) bacterial strains												
*Micrococcus luteus*		25	25	50	2	B	12.5	25	2	B	12.5	25	2	B
*Listeria ivanovii*		25	12.5	25	2	B	50	50	1	B	50	50	1	B
*Staphylococcus aureus*		25	25	50	2	B	12.5	50	4	b	25	50	2	B
		Gram (−) bacterial strains												
*Salmonella enterica*		12.5	12.5	25	2	B	50	50	1	B	50	50	1	B
*Escherichia coli*		12.5	25	50	2	B	50	50	1	B	25	50	2	B
*Pseudomonas aeruginosa*		12.5	25	25	1	B	25	50	2	B	25	50	2	B
		Fungal strains												
*Aspergillus niger*		NA	NA	NA	NA	NA	NA	NA	NA	NA	NA	NA	NA	NA
*Fusarium oxysporum*		NA	12.5	25	2	F	12.5	25	2	F	25	50	NA	NA
		Yeast strain												
*Candida albicans*		12.5	25	50	2	F	25	25	1	F	25	50	NA	NA

**^a^** Ratios MBC/MIC for bacteria and MFC/MIC for fungal and yeast. **^b^** Interpretation: B: bactericidal, b: bacteriostatic, F: fungicidal, f: fungistatic activity. NA: not active.

## Supplementary Materials

Table S1 contains data of monosaccharides and disaccharides arranged by neutral loss in mass spectrometry.
