# Supplementary material for: ESI-MS/MS Analysis of Phenolic Compounds from Aeonium arboreum Leaf Extracts and Evaluation of their Antioxidant and Antimicrobial Activities
Source: Molecules. 2021 Jul 17;26(14):4338. doi: 10.3390/molecules26144338 (PMC8306197; doi:10.3390/molecules26144338)
Supplement: Supplementary file 1 [file molecules-26-04338-s001.zip › molecules-1275598-supplementary.pdf]

Supplementary Materials

Table S1. Monosaccharides and disaccharides arranged by neutral loss in mass spectrometry

		Neutral losses of monosaccharides			
		**Arabinose**	**Rhamnose**	**Glucose**	**Glucuronic acid**
		+132	+146	+162	+176
**Familiar monosaccharides and their neutral losses**		**Neutral losses of glycan with two sugars**			
**Arabinose**	132	264	278	294	308
**Rhamnose**	146	278	292	308	322
**Glucose**	162	294	308	324	338
**Glucuronic acid**	176	308	322	338	352
